# Birth cohort relative to an influenza A virus’s antigenic cluster introduction drives patterns of children’s antibody titers

**DOI:** 10.1371/journal.ppat.1010317

**Published:** 2022-02-22

**Authors:** Andrew F. Brouwer, Angel Balmaseda, Lionel Gresh, Mayuri Patel, Sergio Ojeda, Amy J. Schiller, Roger Lopez, Richard J. Webby, Martha I. Nelson, Guillermina Kuan, Aubree Gordon

**Affiliations:** 1 Department of Epidemiology, University of Michigan, Ann Arbor, Michigan, United States of America; 2 Sócrates Flores Vivas Health Center, Ministry of Health, Managua, Nicaragua; 3 Sustainable Sciences Institute, Managua, Nicaragua; 4 Department of Infectious Diseases, St. Jude Children’s Research Hospital, Memphis, Tennessee, United States of America; 5 Laboratory of Parasitic Diseases, Division of Intramural Research, National Institute of Allergy and Infectious Diseases, National Institutes of Health, Bethesda, Maryland, United States of America; 6 Centro Nacional de Diagnóstico y Referencia, Ministry of Health, Managua, Nicaragua; Johns Hopkins Bloomberg School of Public Health, UNITED STATES

## Abstract

An individual’s antibody titers to influenza A strains are a result of the complicated interplay between infection history, cross-reactivity, immune waning, and other factors. It has been challenging to disentangle how population-level patterns of humoral immunity change as a function of age, calendar year, and birth cohort from cross-sectional data alone. We analyzed 1,589 longitudinal sera samples from 260 children across three studies in Nicaragua, 2006–16. Hemagglutination inhibition (HAI) titers were determined against four H3N2 strains, one H1N1 strain, and two H1N1pdm strains. We assessed temporal patterns of HAI titers using an age–period–cohort modeling framework. We found that titers against a given virus depended on calendar year of serum collection and birth cohort but not on age. Titer cohort patterns were better described by participants’ ages relative to year of likely introduction of the virus’s antigenic cluster than by age relative to year of strain introduction or by year of birth. These cohort effects may be driven by a decreasing likelihood of early-life infection after cluster introduction and by more broadly reactive antibodies at a young age. H3N2 and H1N1 viruses had qualitatively distinct cohort patterns, with cohort patterns of titers to specific H3N2 strains reaching their peak in children born 3 years prior to that virus’s antigenic cluster introduction and with titers to H1N1 and H1N1pdm strains peaking for children born 1–2 years prior to cluster introduction but not being dramatically lower for older children. Ultimately, specific patterns of strain circulation and antigenic cluster introduction may drive population-level antibody titer patterns in children.

## Author summary

The human antibody response to influenza A viruses is complex, in part because these viruses readily mutate to evade our immune systems. Occasional greater genetic changes can establish a new group of viruses, called an antigenic cluster, based on the similarity of our antibody responses. A better understanding of how our antibody responses change over time could improve vaccination strategies around strain selection, vaccination timing, whether yearly vaccination is optimal, and how much variability in vaccination response by age to expect. In this study, we examined how antibody responses change along three dimensions of time, namely age, calendar year, and birth cohort. As expected, antibody titers depended on what years each influenza subtype circulated. We also found that antibody responses to a virus depended on one’s age when a related virus from the same antigenic cluster first circulated, not when that specific virus circulated. We also find evidence young children may have antibodies that cross-react with strains that have not yet circulated, possibly indicating that their immune systems are creating a wide array of antibodies, which could inform possible mechanisms of antigenic seniority. Our results shed new light on the dynamics of antibody titer levels.

## Introduction

The human immunological response to influenza A viruses is comprised of highly complex antibody and cellular responses [1]. In particular, antibodies to influenza surface proteins, especially hemagglutinin (HA), are known to be protective against infection [2]. This immunological response creates evolutionary pressure on the virus, leading to continual antigenic drift, punctuated by larger changes in antigenicity that lead to the formation of antigenic clusters [3]. Antigenic cartography, or mapping, of antigenic data has been one approach used to better visualize the antigenic relatedness of strains, demonstrating which strains form antigenic clusters [4]. There are different approaches to defining antigenic similarity and thus clustering, such as using ferret sera [4, 5] or models of HA sequences [6, 7]. Ultimately, an antigenic cluster should define a set of viruses for which immune recognition is similar.

Our understanding of the immune response to influenza is complicated by the complex interplay of the cross-reactivity of antibodies to related viruses (whether in the same antigenic cluster or not), possible antigenic seniority or imprinting effects, cellular immunity, waning immunity, and other factors [1, 8]. Thus, our understanding of the immunology and correlates of protection against infection or disease remains imperfect, hampering our abilities to understand who is at risk and to develop more effective vaccines against seasonal and pandemic influenza strains.

Infection histories are important for understanding an individual’s antibody response to influenza, as measured, for example, by the hemagglutination inhibition (HAI) assay. An individual’s titer levels depend on the exact timing and set of infections [9, 10], and previous work has explored whether individual infection histories can be inferred from individual longitudinal titers [10–13]. Antibody patterns aggregated at the population level can also help to elucidate underlying antibody dynamics [9, 14], although they can be difficult to interpret, especially in cross-sectional data. We must disentangle the contributions of age, age relative to the time of virus introduction (which is a way of understanding birth cohorts), and patterns of virus circulation across different years to population-average antibody levels for a given virus. In particular, there is a growing interest in understanding drivers and implications of cohort effects for influenza [8, 14–16].

Here, we analyze population-level patterns of HAI titers to seven influenza viruses from two influenza A subtypes—H3N2 and H1N1—in a longitudinal cohort of young adolescents and children, many starting from birth, allowing us to better understand how antibody titer patterns change through infancy and childhood. This analysis will allow us to better understand the role of age, patterns of virus circulation, and viral changes, both between and within subtypes, on antibody responses.

## Methods

### Ethics statement

All studies that provided data for this analysis were approved by the appropriate institutional review boards (at one or more of the Nicaraguan Ministry of Health, University of California Berkeley, and University of Michigan), and parents/guardians of all participants gave written, informed consent to participate and to share data across these studies.

### Data

**Fig 1:**
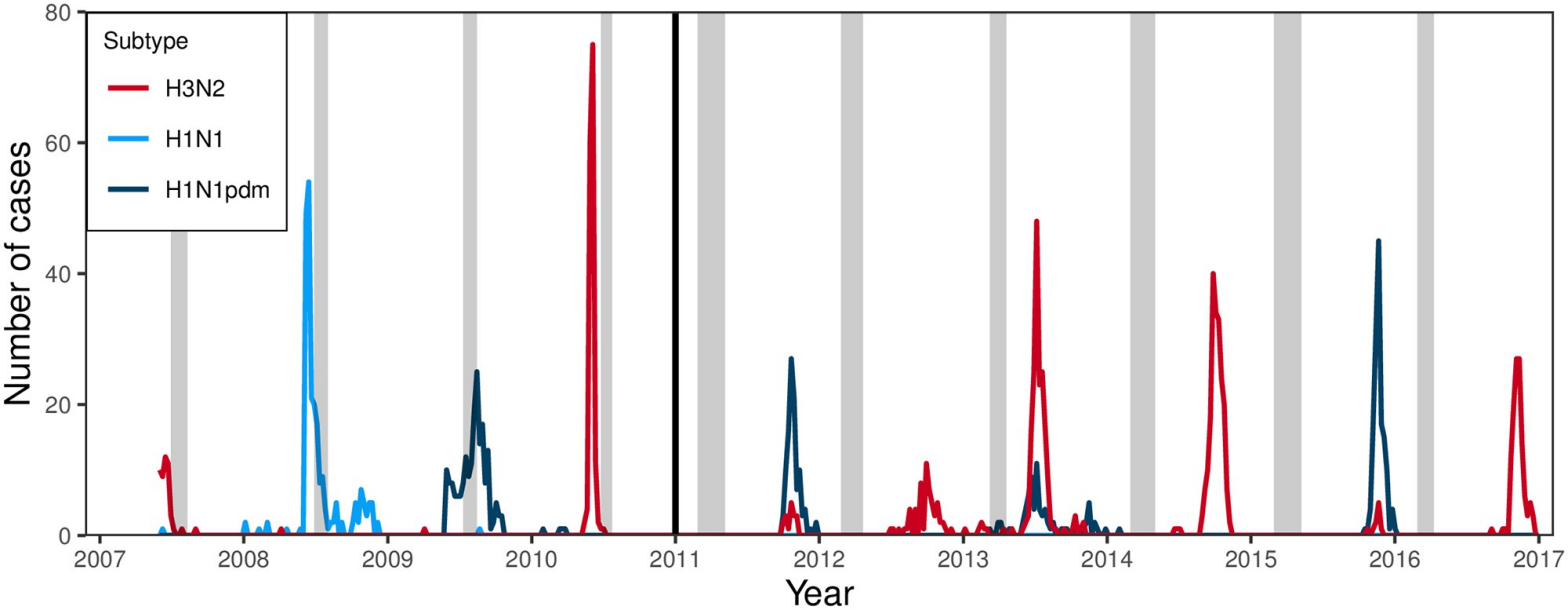
Incidence of influenza A by subtype in the Nicaraguan Influenza Cohort Study (NICS) and Nicaraguan Pediatric Influenza Cohort Study (NPICS). Incidence is defined as RT-PCR-confirmed cases of influenza in study participants presenting at the study health clinic with fever or influenza-like illness. In Nicaragua, influenza A circulation typically peaks mid-year (June/July) or at the end of the year (November/December). Sera samples were collected annually (July or August prior to 2011; March or April in 2011 and after). Grey bars give the period of sera sampling. Incidence before and after 2011 should not be directly compared. The cohort prior to 2011 was larger, but only a randomized subsample of eligible participants were tested, while after 2011 the cohort was smaller, but all eligible participants were tested.

Sera samples were collected from 260 participants enrolled in at least one of three studies in District II of Managua, Nicaragua administered by the Sustainable Science Institute in 2006–2016: the Nicaraguan Influenza Cohort Study (NICS), the Nicaraguan Pediatric Influenza Cohort Study (NPICS), and the Nicaraguan Influenza Birth Cohort Study (NIBCS) [17–19]. The studies initially consisted only of children ages up to age 12, but NPICS expanded up to age 14 in 2013. Enrollment of participants prior to 1 year of age began in 2011. All participants were born in 1995 or later. Sera samples were collected annually (July or August prior to 2011; March or April in 2011 and after). Additional, intermittent samples timed between the annual samples were available for a subset of participants. In Nicaragua, influenza A circulation typically peaks mid-year (June/July) or at the end of the year (November/December). Influenza A incidence by subytpe in the pediatric cohorts (as determined by RT-PCR testing of in swabs of symptomatic study participants) and the timing of the annual sera sample collection is given in Fig 1. Additional details on the study site, population, and design may be found in [17–19]. In total, 1,589 samples were analyzed. A table of number of samples by age and year is included in Table A in S1 Appendix.

### Laboratory assays

HAI assays were performed for four H3N2 influenza vaccine strains (A/Wisconsin/67/2005, A/Perth/16/2009, A/Victoria/361/2011, A/Texas/50/2012, the former belonging to the WI05 antigenic cluster and latter three belonging to the same PE09 antigenic cluster [4, 20–22]) and three H1N1 influenza vaccine strains (seasonal A/Solomon Islands/3/2006 and pandemic A/California/7/2009 and A/Michigan/45/2015, the last two belonging to the same CA09 antigenic cluster). Serological testing was performed at the University of Michigan. Participants with undetectable titers were assumed to have a titer of 1:5. Molecular testing for influenza was performed at the National Virology Laboratory of the Centro Nacional de Diagnóstico y Referencia, which is an WHO National Influenza Center and part of the Nicaraguan Ministry of Health.

### Age–period–cohort modeling

**Fig 2:**
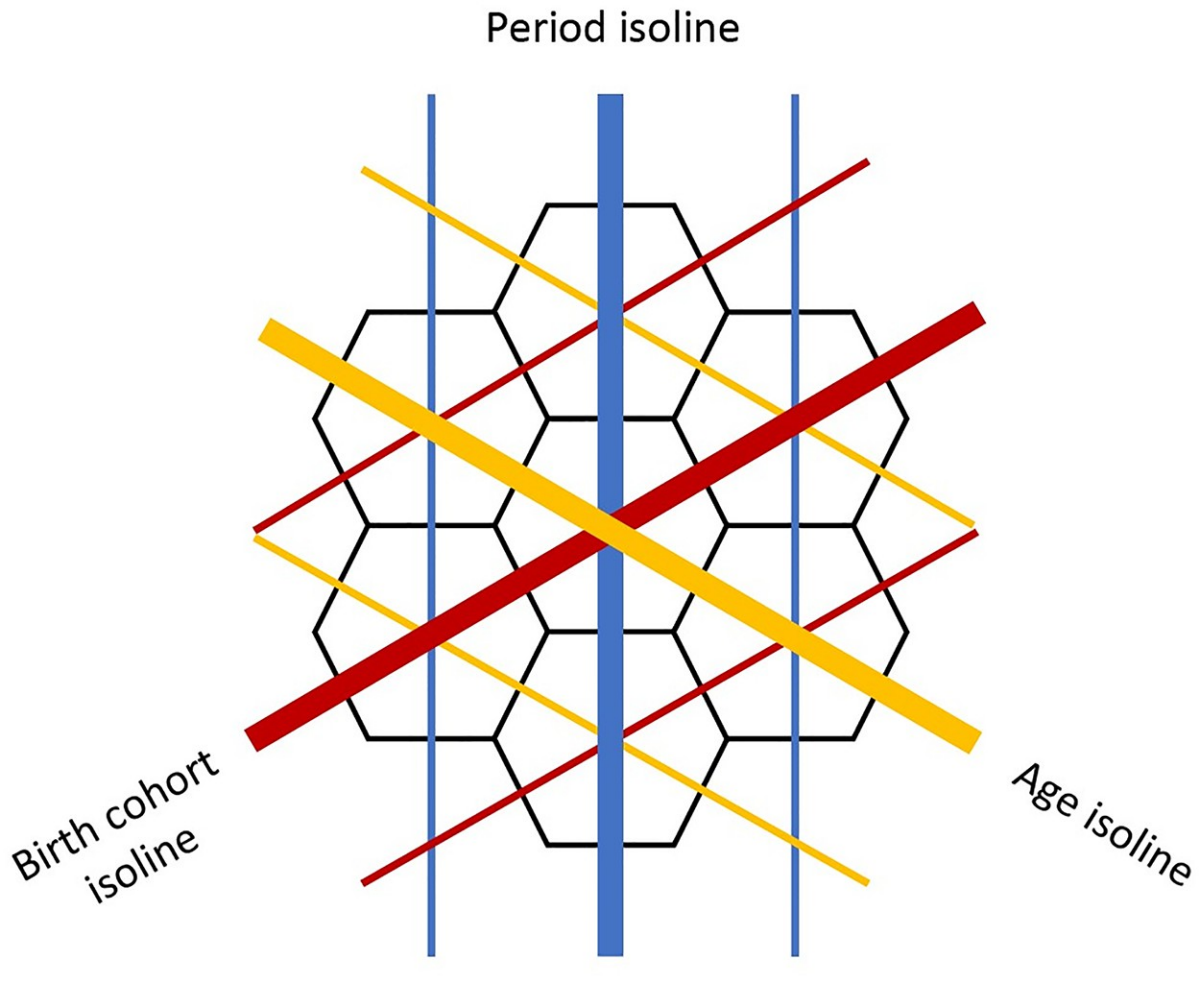
Hexamaps. Hexamaps visualize longitudinal population survey data by distinguishing by age, calendar year (period), and birth cohort along each of three axes. All points along an isoline share the same value one of the temporal characteristics.

Age–period–cohort (APC) modeling is a generalized linear modeling technique used to distinguish between different effects of time (age, period or calendar year, and cohort or birth year) in longitudinal data [23–26]. Hexamaps offer one way of visualizing the underlying data, with the three axes representing age, period, and birth cohort (Fig 2) [27]. The general formulation of an APC model for HAI titer *y* as a function of age *t*_*a*_, period *t*_*p*_, and cohort *t*_*c*_ is

$$\begin{array}{c} \text{log}\;y=\beta_{0}+\beta_{a}\left(t_{a}\right)+\beta_{p}\left(t_{p}\right)+\beta_{c}\left(t_{c}\right) \end{array}$$ (1)

For each subtype, we considered both strain-specific models and subtype-specific models, namely, for strain *i*_*j*_ in influenza subtype *i*, we defined the strain-specific model

$$\begin{array}{c} \text{log}\;y_{i_{j}}=\beta_{0,i_{j}}+\beta_{a,i_{j}}\left(t_{a}\right)+\beta_{p,i_{j}}\left(t_{p}\right)+\beta_{c,i_{j}}\left(t_{c}\right), \end{array}$$ (2)

and the subtype-specific model

$$\begin{array}{c} \text{log}\;y_{i_{j}}=\beta_{0,i_{j}}+\beta_{a,i}\left(t_{a}\right)+\beta_{p,i}\left(t_{p}\right)+\beta_{c,i}\left(t_{c}\right). \end{array}$$ (3)

The difference between these models is whether the time effects can be considered fixed for all strains in a subtype (*β* does not depend on *j*), or whether each strain requires its own time effects (*β* depends on *j*). In the subtype-specific models, there are several different ways to specify the cohort *t*_*c*_. We considered three here: birth year, age at strain introduction, and age in a strain-specific year, which we interpret as the time of the introduction of a genetically similar virus, i.e., a virus in the same antigenic cluster.

In this analysis, *β*_*a*_ and *β*_*c*_ were cubic B-splines with 3 and 4 degrees of freedom, respectively, and *β*_*p*_ was a step function taking different values for each calendar year. Because *t*_*a*_ = *t*_*p*_ − *t*_*c*_, the parameters of (1) are not identifiable without further assumptions. In this analysis, we considered models with only two of *t*_*a*_, *t*_*p*_, and *t*_*c*_ at a time.

We compared models using a variety of model metrics, including *R*^2^, root mean squared error, negative log-likelihood, and the Schwarz Information Criterion (SIC). The SIC, also known as the Bayesian Information Criterion (BIC), is a measure of model fit with a penalty for the number of parameters, which controls overfitting, and it is particularly helpful here in comparing whether the added parameters of the strain-specific models compared to the subtype-specific models are justified. The SIC is defined as log(n)·k-2logL, where *n* is the sample size, *k* is the number of model parameters and L is the model likelihood. The model with the lowest SIC value can be thought of as the simplest model that fits the data well.

## Results

### Participant and sample statistics

Characteristics of the participants are summarized in Table 1. Of the 260 participants, 55% (142) were recruited prior to their first birthday. Of those not recruited prior to age 1, the median age of recruitment was 3, with a range of 1 to 11. At participants’ baseline visits, 62% (162) exhibited titers of at least 1:20 to at least one of the four H3N2 strains (including 57% (81) of participants recruited prior to 1 year of age), and 34% (88) exhibited titers to at least one of the three H1N1 strains (including 26% (37) participants recruited prior to 1 year of age). The participants had a median of 5 analyzed samples, with a range of 1 to 19 samples (including both annual and intermittent samples). There were 53 confirmed (e.g., by vaccine card) and 39 probable (e.g., self-reported and consistent with clinic administration dates of vaccine administration) influenza vaccinations among 63 participants within the span of the data. Most vaccinations occurred in May or June of 2012, 2014, or 2015, after the sera sampling period for that year. We did not exclude these individuals from the analysis but instead interpret the antibody titer results, particularly period effects in these years, as potentially being impacted by vaccination rather than infection, if any effect of vaccination on antibody titers was still detectable by the sera sampling period the following year.

**Table 1:** Characteristics of the study cohort at time of each participant’s first sera sample.

Characteristics	% (N) or mean (range)
Age	1.8 (0–11)
Sex	
Female	45% (116)
Male	55% (144)
Ever received influenza vaccine	
Yes	24% (63)
No	76% (197)
Number of household residents	8.1 (1–30)
Mother is literate	
Yes	76% (198)
No	3% (9)
Unknown	20% (53)
Water available inside the home	
Yes	54% (140)
No	26% (68)
Unknown	20% (52)
Home has dirt floor	
Yes	12% (32)
No	66% (172)
Unknown	22% (56)
Cook with firewood in the home	
Yes	11% (29)
No	50% (130)
Unknown	39% (101)
Animals in the home	
Yes	35% (92)
No	27% (71)
Unknown	37% (97)

### Data visualization

**Fig 3:**
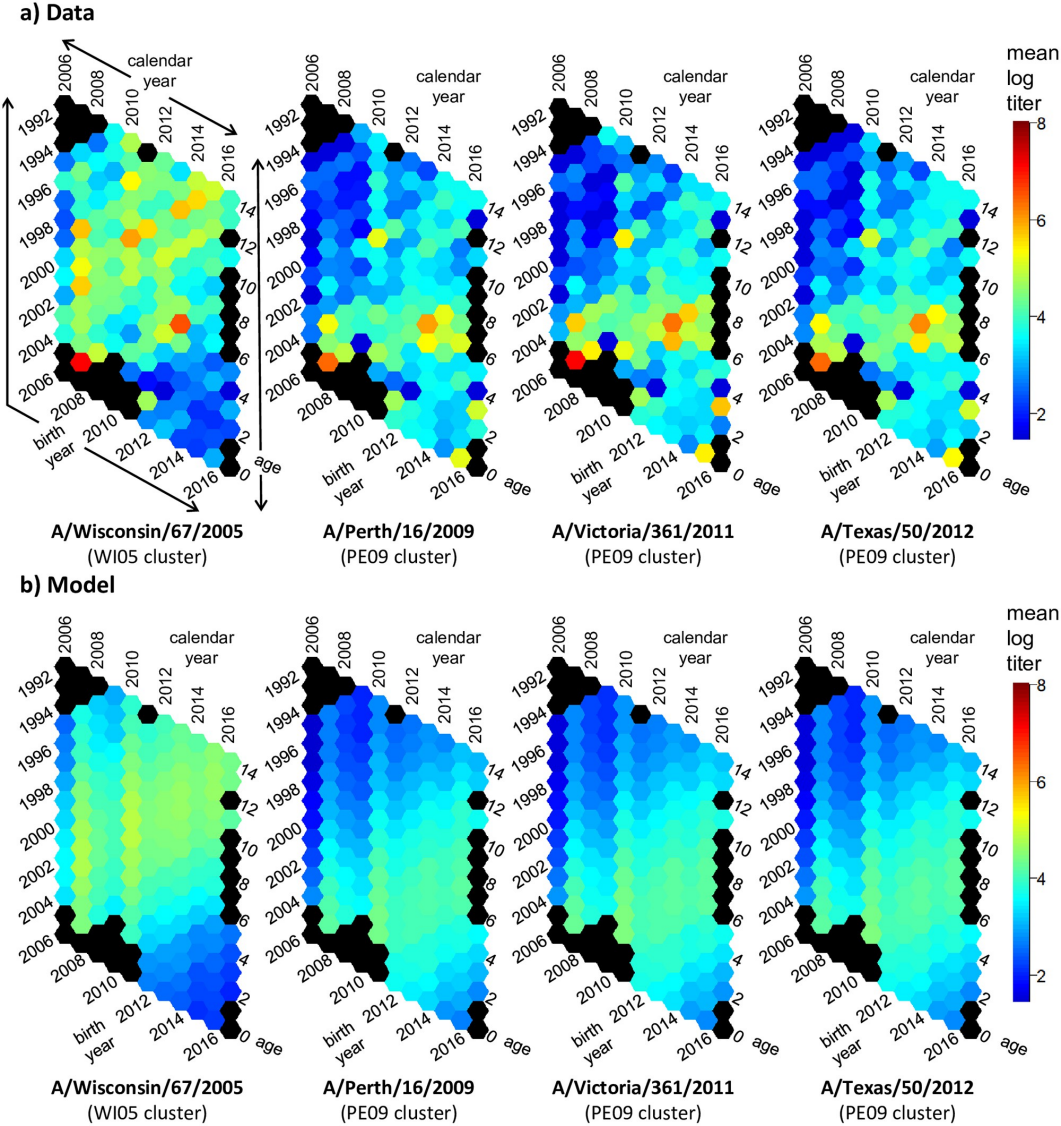
H3N2 hexamaps for HAI titers. a) Hexamaps visualizing H3N2 HAI mean log titers as a function of age, calendar year, and birth year in the data. b) Corresponding hexamaps for the mean log titers predicted by the H3N2 subtype-specific model. Black hexagons represent points with no participants.

**Fig 4:**
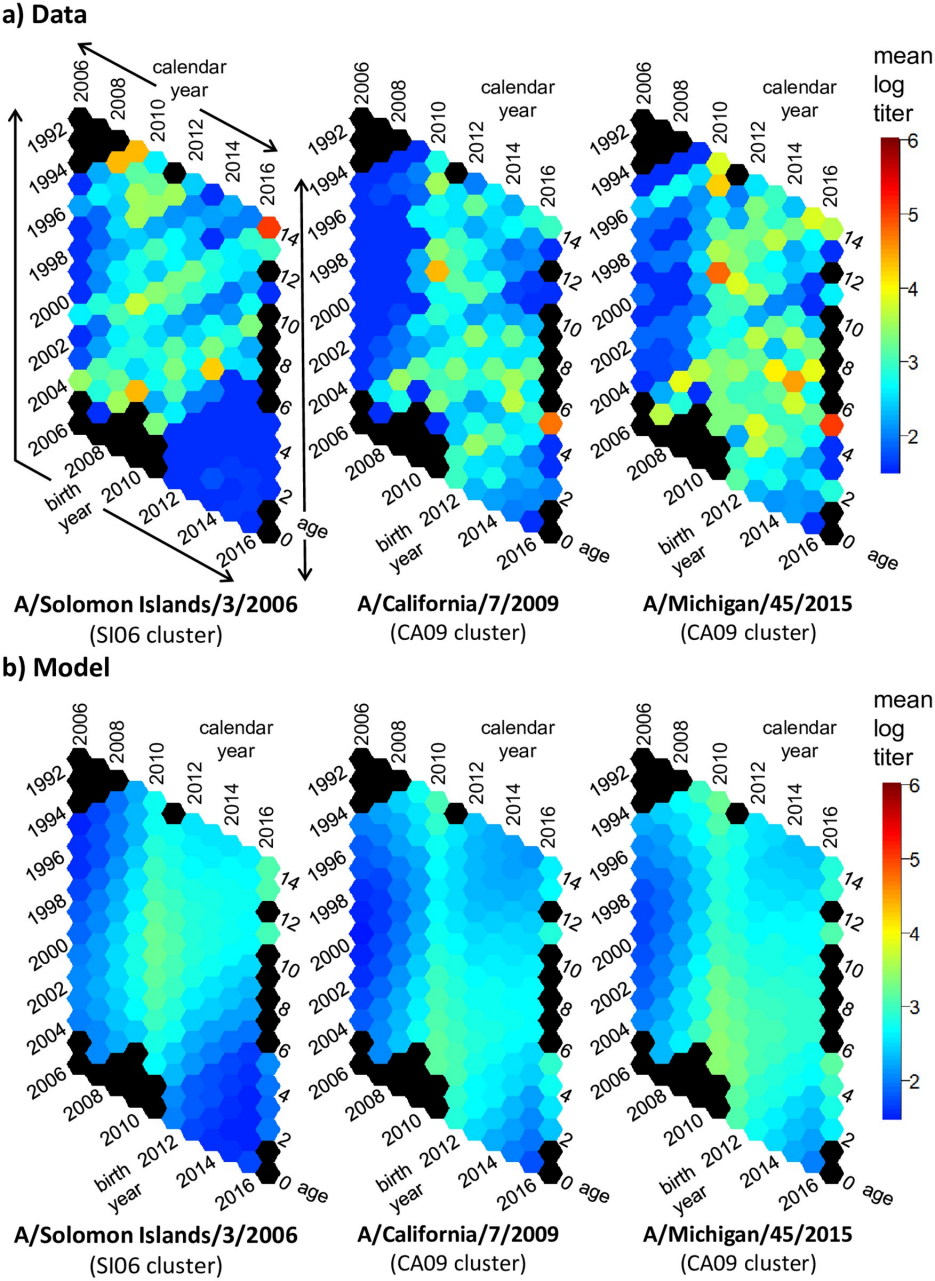
H1N1 hexamaps for HAI titers. a) Hexamaps visualizing H1N1 HAI mean log titers as a function of age, calendar year, and birth year in the data. b) Corresponding hexamaps for the mean log titers predicted by the H1N1 specific model with separate time effects for seasonal and pandemic strains. Black hexagons represent points with no participants.

We present the mean log titers as hexamaps illustrating age, calendar year, and birth cohort patterns for the H3N2 strains in Fig 3a and the H1N1 strains in Fig 4a. The sample size underlying each hex is given in Fig A in S1 Appendix. Individual log-titers are presented in Figs B and C in S1 Appendix; correlations between an individual’s titers to each virus is given in Fig D in S1 Appendix. In these hexamaps, there were clear patterns in each plot by year of sample collection and participant birth cohort; patterns by age, if any, are less apparent. In the H3N2 data, samples collected in 2007 and 2010 have noticeably high titers. This result is not surprising, given that collection of the samples in 2007 and 2010 was directly after H3N2 epidemics (Fig 1). Titers to A/Wisconsin/67/2005 began to decrease around the 2008 birth cohort. Titer patterns to A/Perth/16/2009, A/Victoria/361/2011, and A/Texas/50/2012 were nearly identical, with higher titers in the samples collected after 2009 for birth cohorts prior to 2004 and higher titers in those collected after 2007 for birth cohorts 2004 and later. Seasonal H1N1 strains stopped circulating in 2009 with the introduction of H1N1pdm viruses, and the strongest effects for A/Solomon Islands/3/2006 were seen by birth cohort, with very low titers in birth cohorts 2009 and later. For the H1N1pdm strains A/California/7/2009 and A/Michigan/45/2015, titers were low prior to 2009, except for participants in the 2005–06 birth cohorts, who, surprisingly, had titers prior to 2009. (Note: there were no participants in the study at the time who were in the 2007–08 birth cohorts). These pre-2009 titers were not driven by single individuals. Three of the 7 participants who were age 2 at sample collection in 2007 and 4 of the 8 participants who were age 2 in 2008 had positive titers to A/California/7/2009. Titers to the H1N1pdm strains decreased in the birth cohorts after 2012.

### Model comparisons and changes in titer by cohort

**Fig 5:**
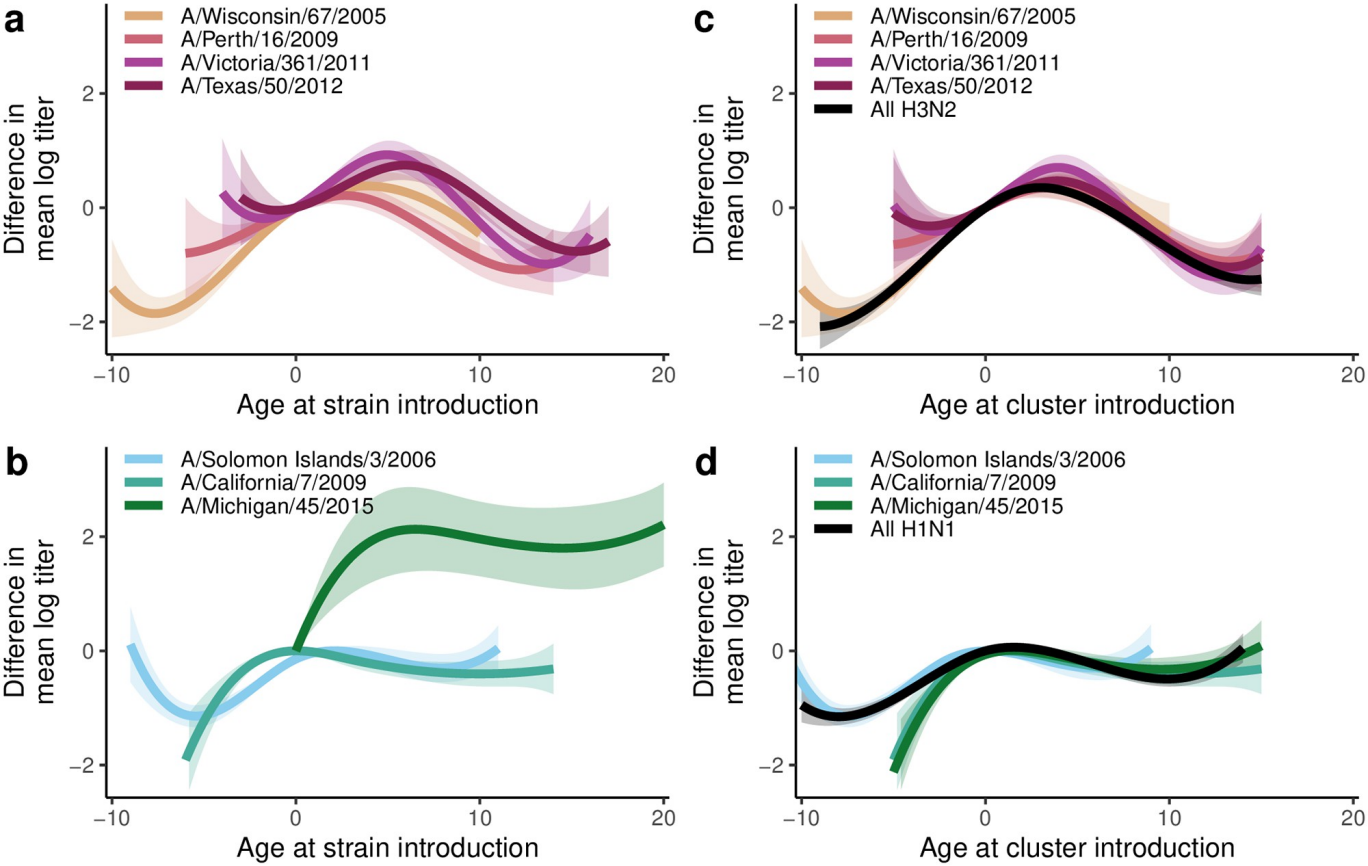
Cohort effects on HAI titers. a) Difference in mean log titer in the H3N2 strain-specific models by age at strain introduction. b) Difference in mean log titer in the H1N1 strain-specific models by age at strain introduction (note: for A/Michigan/45/2015 we linearly impute the effect to age 0). c) Difference in mean log titer in the H3N2 strain-specific models and the H3N2 subtype model by age at antigenic cluster introduction. d) Difference in mean log titer in the H1N1 strain-specific models and the H1N1pdm model by age at antigenic cluster introduction. In each plot, the difference in mean log titer is relative to age 0 at strain or cluster introduction, and the bands give the 95% confidence intervals.

As expected from the patterns identified in the data visualization, we find that the strain-specific models are best described by period–cohort models (as determined by a variety of model metrics given in Tables B and C in S1 Appendix). In Fig 5a and 5b, we plot the cohort effects for each model as a function of age at the time of the corresponding strain introduction. We see that, as a function of age at strain introduction, the effects have similar shapes within subtype but are offset. In Fig 5c and 5d, we control for that offset. Specifically, rather than age at strain introduction, we plot the cohort effects as a function of age in a strain-specific year: 2005 for A/Wisconsin/67/2005, 2010 for A/Perth/16/2009, A/Victoria/361/2011, and A/Texas/50/2012, 2004 for A/Solomon Islands/3/2006, and 2009 for A/California/7/2009 and A/Michigan/45/2015. We interpret these years as the time that a virus of the same antigenic cluster first circulated locally. We also observe that titers to H3N2 viruses appear to increase and decline symmetrically by cohort, peaking in children born approximately 3 years before the virus’s antigenic cluster introduction, while the titers to the H1N1 strains, which peak in children born 1–2 years prior to cluster introduction, may also be higher for older children. Only one cluster change was observed for each of H3N2 and H1N1, and we estimate that the previous cluster was dominant for about 5 years (2005–2010 for WI05, 2004–2009 for the cluster prior to CA09). These shapes reflect averages over the study period for individuals in each birth cohort (i.e., averaging individual’s trajectories) adjusting for calendar year effects. Analogous figures giving the raw mean log titers are given in the Fig E in S1 Appendix and bootstrap cohort effects for the subtype-specific models are given in Fig F in S1 Appendix.

Analysis of the children included prior to their first birthday suggests that these patterns are largely driven by when children have their first infection and how that depends on time since cluster introduction. Specifically, the fraction of children who have antibodies (i.e., HAI titers greater than 1:5) prior to their first birthday (likely from early-life infection, as maternal antibodies would likely have waned by the time sera was collected, at least 6 months after birth [28]) decreased with time since cluster introduction (Fig G in S1 Appendix). Children with early-life antibodies may or may not maintain them, leading to slight waning of the population average over the next 5 years (Fig H in S1 Appendix). Children who did not have an infection prior to their first birthday catch up over those 5 years, so that the two groups become largely indistinguishable. In these data, this merging takes 2–4 years for H3N2 strains and 4–5 years for H1N1 strains. Although, the cohort of children enrolled prior to age 1 does not span the full data (as they belong to birth cohorts of 2010 and later), these results suggest that this cohort effect on the population average HAI titers may be driven in part by the age at first infection increasing with time since cluster introduction.

Looking at the full sample patterns reinforces the importance of a child’s birth year relative to the time of antigenic cluster introduction (Fig I in S1 Appendix). Children in the birth cohorts shortly preceding the antigenic cluster introduction (those with the highest titers overall) had cross-reactive antibodies prior to circulation of the first virus in the new antigenic cluster. Children in earlier birth cohorts have lower titers to the newer cluster viruses overall because they initially begin with lower titers to those viruses but then have titers boosted around the time of circulation. Children born while strains of that virus’s antigenic cluster are circulating appear to maintain relatively high levels of HAI antibodies cross-reactive to that strain, at least as long as strains in that cluster are circulating. Children born after strains in a given antigenic cluster have stopped circulating had few cross-reactive HAI antibodies to strains in that cluster.

In Table 2, we compare the strain- and subtype-specific models with period and cohort effects for the different definitions of cohort (lower/more negative values indicate better fits) using the SIC (a broader comparison of models and model metrics is given in Tables B and C in S1 Appendix). For the strain-specific models, changing the cohort definition is equivalent to changing the referent year, so there is no change to the model SIC. When two strains share period and cohort effects, however, as in the subtype-specific models, the specification of the cohort effect does matter. Here, we see that the subtype-specific H3N2 model with cohort defined as age at cluster introduction is preferred by the SIC over the strain-specific model. This result means that we do not need strain-specific period and cohort parameters to explain the data: viruses of the same subtype have similar period and cohort patterns. Similarly, the subtype-specific model is preferred for the H1N1 strains. Modeled mean log titers for the H3N2 subtype-specific model and the H1N1 subtype-specific model, each with cohort defined as age at cluster introduction, are plotted in Figs 3b and 4b. The overall H3N2 and H1N1 cohort effects are included in Fig 5c and 5d.

**Table 2:** Comparison of period–cohort models for the H3N2 and H1N1 strains.

Model	Cohort definition		
	Birth year	Age at strain introduction	Age at cluster introduction
H3N2			
Strain-specific	Ref.	Ref.	Ref.
Subtype-specific	115.5	-266.0	-346.0
H1N1			
Strain-specific	Ref.	Ref.	Ref.
Subtype-specific	58.7	-21.4	-136.3

Values are Schwarz Information Criterion (SIC) relative to the strain-specific SIC. H3N2 strain specific SIC: 24772.2. H1N1 strain-specific SIC: 15849.3.

### Changes in titers by period

**Fig 6:**
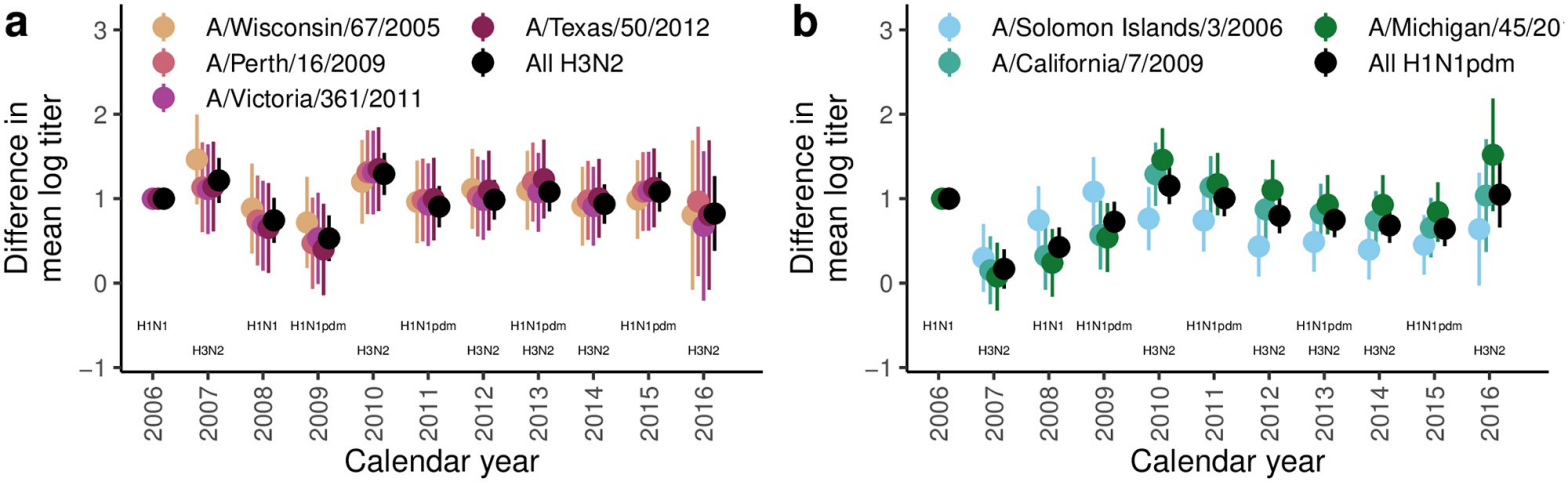
Period (calendar year) effects on HAI titers. Difference in mean log titer relative to 2006 for a) the H3N2 strain-specific and subtype-specific models and b) the H1N1 strain-specific and seasonal vs pandemic strain models. In each plot, the bars give the 95% confidence intervals.

The period effects for the strain-specific, H3N2 subtype-specific, and H1N1 seasonal vs pandemic strain models are shown in Fig 6. As seen in the hexamaps, 2007 and 2010 are years of high H3N2 titers, and these correspond to years when H3N2 circulated (Fig 1). After 2010, the correlation between larger mean log titers and circulation of H3N2 is less pronounced, possibly because of the shift in protocol, starting in 2011, to collecting samples in March/April (on average 6 months after the previous peak) instead of July/August (typically directly after or during circulation). For H1N1, 2009 is a high titer year for the seasonal strain, while 2010 is the high titer year for the pandemic strains, likely because the 2009 samples were taken during the early phases of the H1N1pdm epidemic (Fig 1). Altogether, the seasonal H1N1 period effect (relative to 2006) is greater than the pandemic H1N1 period effect before 2010 and lesser afterward.

## Discussion

In this study, we find evidence that population-level antibody patterns to an H3N2 or H1N1 influenza A virus in children depend neither on age, per se, nor on age at the time that virus first circulated, but instead on the age when a strain from that virus’s antigenic cluster first circulated locally. The patterns of titers to A/Perth/16/2009, A/Victoria/361/2011, and A/Texas/50/2012, which are all in the Perth 2009 cluster, are consistent with circulation of an H3N2 strain in 2010 that was antigenically distinct from previously circulating viruses [29]. Indeed, phylogenetic analysis indicates that the 2007 strain was similar to A/Brisbane/10/2007, a virus in the WI05 cluster [4] and that the 2010 strain similar to A/Perth/16/2009, from the PE09 cluster (Fig J in S1 Appendix). For H1N1, the same pattern arises, with titers to A/California/7/2009 and A/Michigan/45/2015 being driven by age in 2009, the year that H1N1pdm first circulated in Nicaragua. Previous work has used continuous functions of time between virus circulation or of antigenic distance to approximate cross-reactivity [9, 10]. Our work suggests that cross-reactivity may be closer to a step function that depends on larger antigenic changes and antigenic clustering.

Our results shift the emphasis from the response to specific strains to the response to viruses of the same antigenic clusters and from a child’s age to the time patterns of local circulation. Previous studies have focused on the impact of age at strain introduction and estimated that antibody titers to H3N2 viruses are highest in children who were born 4–10 years before the virus was first isolated [12, 14, 20]. Our findings suggest that antibody titers to an H3N2 are highest in children who were born 3 years prior to local circulation that virus’s antigenic cluster. Our results are not a contradiction of the prior work but instead offer a different perspective on how we might view trends in antibody titers. Moreover, the specific epidemiological context and typical patterns of circulation likely determine the specific shape of the cohort effects, which may not be generalizable. We attribute the cohort effect observed here in part to the dynamics of first infection because the fraction of children with antibodies prior to their first birthday drops off after cluster introduction, suggesting that the time to an individual’s first H3N2 infection increases with time since the introduction of a new antigenic cluster. The dynamics of infection in adults may be relevant here—if adults gain immunity shortly after an antigenic cluster introduction, their infants may have a reduced chance of exposure if born after that antigenic cluster first circulated. The cohort effect may also be a result of age-related antibody specificity or affinity. We find evidence of cross-reactive antibodies in children under 4 to viruses that are part of antigenic clusters that have not yet circulated. For example, we see titers to A/California/7/2009 and A/Michigan/45/2015 in samples collected *prior* to 2009 (Fig 4a). We also see higher antibody titers to A/Perth/16/2009, A/Victoria/361/2011, and A/Texas/50/2012 in this same subsample prior to the circulation of that cluster in 2010, after which we see titers rise more among the broader population (Fig 3a). One explanation is that the immune response of young children is more broadly reactive, creating a wide array of lower-affinity antibodies, some of which may cross-react to future strains [4, 30]. If young children are producing antibodies that are more broadly reactive and are ready to be boosted upon cluster change, that would result in the highest antibodies in those born a few years prior to cluster introduction.

We find that the cohort pattern for H1N1 was somewhat different from that of H3N2: antibodies appeared to peak for those born in the year of circulation of the antigenically distinct strain but not be dramatically lower for children from earlier cohorts. This pattern may be driven by the relatively little antigenic change in the H1N1pdm strains and may not hold over longer periods or periods with more antigenic drift. This pattern may also be related to the greater frequency of H3N2 circulation compared to H1N1 (which may itself be related to speed of genetic drift). We found that children that did not have early-life antibodies took longer to gain titer levels similar to those that did have early life antibodies for H1N1 strains (4–5 years) compared to H3N2 strains (2–4 years).

The question of antigenic seniority, its mechanisms, and its impacts on antibody dynamics has remained important to the field [30, 31]. Our work suggests that a clear and definitive understanding of antigenic seniority would require the observation of many different birth cohorts each with longitudinal data spanning multiple antigenic cluster introductions. Nevertheless, we do see some evidence of the impacts of antigenic seniority in these data. First, we see boosting of titers to strains from previous clusters when strains in later clusters circulate. For example, titers to A/Wisconsin/67/2005, a member of the Wisconsin 2005 antigenic cluster, were boosted in 2010 even though the circulating viruses at the time were part of the Perth 2009 antigenic cluster (Fig 3a). More broadly, we see that the population-level average titers to a strain are highest and generally show little waning for those born prior to the introduction of the cluster that strain belongs to (Fig I in S1 Appendix). These results are consistent with continual boosting of titers to strains in the same cluster that one was exposed to early in life.

Our results regarding the importance of antigenic cluster introduction, while persuasive for these data, should be viewed as suggestive rather than conclusive. We only observed a limited number of strains and antigenic cluster changes over the study time period, so the generalizability of our results more broadly will have to be tested in future work. Additionally, one challenge of interpreting and applying this work is the difficulty in defining antigenic clusters, particularly as timing of antigenic clusters may differ regionally. One approach to inferring antigenic clustering is the use statistical or computational models to predict clustering based on HA sequences [6, 7]. These methods have shown promise, but it is not always clear when sequence-based differences will translate into functional antigenic differences. A standard approach to developing more functional relationship uses ferret sera [4, 5], which has the advantage of measuring actual cross-reactivity. However, antigenic maps derived from ferret sera do not always match the patterns seen in humans [4]. The use of human sera would seem to be a gold standard, but human sera are challenging to use because responses vary with past exposure. Ultimately, more work is needed as a field to develop a consensus understanding of influenza A H3N2 and H1N1 antigenic clusters and to continuously characterize newly circulating strains. Our analysis, which uses antigenic clusters as defined by previous work, has revealed limitations of our existing understanding of antibodies and cross-reactivity; future work will likely determine better measures of the functional relationship between viruses. The SIC metric here justified the use of subtype-specific models over strain-specific models for these data, but future work may suggest that the more complex models are needed in other contexts.

The strengths of this analysis include data supplied by the large, long-running, longitudinal cohort studies of children, many of whom have been included since birth. Previous studies have considered cross-sectional [9, 10, 14] or limited longitudinal [10, 12] samples. The current age–period–cohort analysis limits how much we can infer about underlying mechanisms, such as boosting, cross-reactivity, and waning, or about individual titer trajectories [9, 10], but the longitudinal design will allow us address these in future work. Finally, previous, similar studies have also focused only H3N2, making the inclusion of H1N1 here an important advance, though some of our inferences regarding H1N1 are limited because seasonal H1N1 stopped circulating at the study site and as the H1N1pdm strains had limited antigenic change.

## Conclusion

Our work demonstrates that patterns of antibody titers in children and young adolescents may depend on the timing of antigenic cluster changes (rather than that of individual virus introduction) and local circulation. We also find evidence that young children may create a wide array of lower-affinity antibodies, which may cross-react with future strains and which may be relevant to the mechanism of antigenic seniority. Ultimately, a more robust and continually updated understanding of antigenic clusters is needed to understand patterns of immunity and predict vaccine effectiveness.

## Supporting information

S1 Data Supporting data. Age–period–cohort data for 1589 sera samples with HAI titers to four H3N2 viruses, one seasonal H1N1 virus, and two pandemic H1N1 viruses.
(CSV)

S1 Code Supporting code. R code that generates the main text figures using S1 Data.
(R) Click here for additional data file.

S1 AppendixSupporting information.Influenza A circulation and serum collection during the study period, the number of samples included by age and year, a comparison of APC models, the raw mean log titers by cohort, several figures developing an interpretation of the population-level cohort effects, and a phylogenetic tree for H3N2 influenza viruses circulating in Nicaragua in 2007 and 2010. **Fig A**. **Number of sera samples by year of collection and participant age**. The color scheme is linear in the log of the sample size to better show the range of variation. **Fig B**. **H3N2 log titers and mean log titers by birth cohort and calendar year**. Individual log titers are jittered to avoid overlaps. a, b) A/Wisconsin/67/2005; c, d) A/Perth/16/2009; e, f) A/Victoria/361/2011; g, h) A/Texas/50/2012. **Fig C**. **H1N1 log titers and mean log titers**. Individual log titers are jittered to avoid overlaps. a, b) A/Solomon Islands/3/2006; c, d) A/California/7/2009; e, f) A/Michigan/45/2015. **Fig D**. **Correlation of influenza A titers by virus within individuals**. **Fig E**. **Cohort effects in the data**. a) Mean log titer of the H3N2 strains by age at cluster introduction. b) Mean log titer of the H1N1 strains by age at cluster introduction. **Fig F**. **Bootstrap cohort effects for a) H3N2 and b) H1N1**. Individual bootstrap estimates are in grey, and the estimate for the original data set is in black. **Fig G**. **Fraction of children enrolled prior to age 1 who had antibody titers to the given strain as a function of the time since cluster introduction**. **Fig H**. **Population-level average mean log antibody titer trajectories**. Trajectories for children enrolled prior to age 1, distinguishing between those who had antibodies to the given strain prior to age 1 and those that did not. **Fig I**. **Mean log titer in each year for each strain, stratifying the population by birth cohort relative to the change in antigenic cluster of the circulating virus**. Red indicates those born more than one antigenic cluster before the given strain’s cluster, purple indicates those born in the antigenic cluster just prior to the given strain’s cluster, dark blue indicates those born in years the given strain’s cluster was circulating, and light blue indicates those born in years after the given’s strains cluster was no longer circulating. **Fig J**. **Maximum likelihood tree of H3 proteins, 2005–10**. Nicaraguan viruses are in red, US viruses are in light green, and vaccine viruses are in blue. The Nicaragua strains from 2007 are BR07-like, and those from 2010 are PE09-like. **Table A**. **Number of sera samples by year of collection and participant age**. **Table B**. **Comparison of APC models for H3N2 log-titers**. Models are compared by degree of freedom (df), *R*^2^, root mean squared error ($\sqrt{\text{MSE}}$), negative log-likelihood (NLL), number of parameters (*N*_par_), and Schwarz Information Criterion (SIC). In the strain specific models, all specifications of cohort are equivalent. **Table C**. **Comparison of APC models for H1N1 log-titers**. Models are compared by degree of freedom (df), *R*^2^, root mean squared error ($\sqrt{\text{MSE}}$), negative log-likelihood (NLL), number of parameters (*N*_par_), and Schwarz Information Criterion (SIC). In the strain specific models, all specifications of cohort are equivalent.(PDF)
