# Supplementary material for: Birth cohort relative to an influenza A virus’s antigenic cluster introduction drives patterns of children’s antibody titers
Source: PLoS Pathog. 2022 Feb 22;18(2):e1010317. doi: 10.1371/journal.ppat.1010317 (PMC8896668; doi:10.1371/journal.ppat.1010317)
Supplement: S1 Appendix — Influenza A circulation and serum collection during the study period, the number of samples included by age and year, a comparison of APC models, the raw mean log titers by cohort, several figures developing an interpretation of the population-level cohort effects, and a phylogenetic tree for H3N2 influenza viruses circulating in Nicaragua in 2007 and 2010. Fig A. Number of sera samples by year of collection and participant age. The color scheme is linear in the log of the sample size to better show the range of variation. Fig B. H3N2 log titers and mean log titers by birth cohort and calendar year. Individual log titers are jittered to avoid overlaps. a, b) A/Wisconsin/67/2005; c, d) A/Perth/16/2009; e, f) A/Victoria/361/2011; g, h) A/Texas/50/2012. Fig C. H1N1 log titers and mean log titers. Individual log titers are jittered to avoid overlaps. a, b) A/Solomon Islands/3/2006; c, d) A/California/7/2009; e, f) A/Michigan/45/2015. Fig D. Correlation of influenza A titers by virus within individuals. Fig E. Cohort effects in the data. a) Mean log titer of the H3N2 strains by age at cluster introduction. b) Mean log titer of the H1N1 strains by age at cluster introduction. Fig F. Bootstrap cohort effects for a) H3N2 and b) H1N1. Individual bootstrap estimates are in grey, and the estimate for the original data set is in black. Fig G. Fraction of children enrolled prior to age 1 who had antibody titers to the given strain as a function of the time since cluster introduction. Fig H. Population-level average mean log antibody titer trajectories. Trajectories for children enrolled prior to age 1, distinguishing between those who had antibodies to the given strain prior to age 1 and those that did not. Fig I. Mean log titer in each year for each strain, stratifying the population by birth cohort relative to the change in antigenic cluster of the circulating virus. Red indicates those born more than one antigenic cluster before the given strain’s cluster, purple indicates th [file ppat.1010317.s003.pdf]

S1 Appendix for *Birth cohort relative to an influenza A virus's antigenic cluster introduction drives patterns of children's antibody titers*

Number of samples by age and year

In the Table A, we provide the breakdown of number of samples by participant age and year of collection. This data is presented in Fig A using a hexamap to facilitate comparison to Fig 3–4.

Table A: Number of sera samples by year of collection and participant age.

Age	Year										
	2006	2007	2008	2009	2010	2011	2012	2013	2014	2015	2016
0	0	0	0	0	0	31	20	26	50	5	0
1	0	1	0	0	0	2	27	18	42	42	0
2	11	7	8	0	2	5	3	48	46	71	5
3	11	10	3	1	4	4	11	26	46	59	1
4	10	11	11	3	9	5	8	9	25	41	1
5	6	10	9	10	4	7	11	8	10	27	4
6	7	6	10	10	19	11	6	5	5	9	0
7	5	7	5	11	13	12	10	14	7	3	0
8	7	4	6	4	14	15	16	8	8	10	0
9	5	7	4	7	5	10	12	14	9	14	0
10	6	5	7	4	10	5	10	17	15	8	0
11	2	6	5	8	4	7	7	13	15	19	2
12	0	2	6	5	7	5	12	4	12	13	0
13	0	0	2	7	6	10	6	11	5	11	2
14	0	0	0	1	9	0	10	6	6	5	2

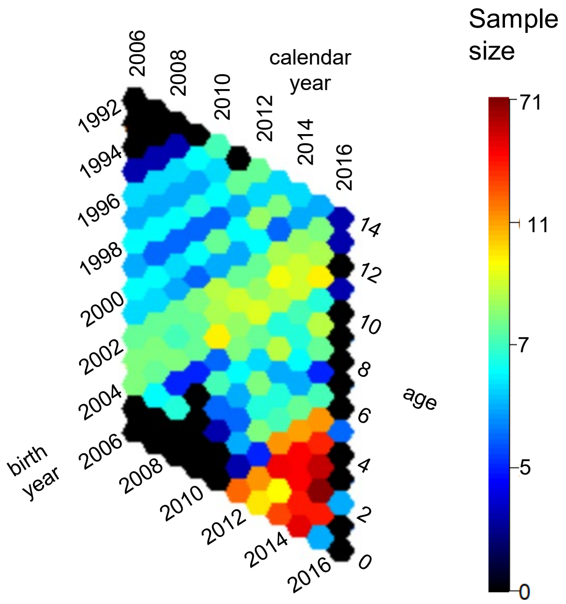

Figure A: **Number of sera samples by year of collection and participant age.** The color scheme is linear in the log of the sample size to better show the range of variation.

Raw titers

In Figures B and C, we plot raw log titers (jittered to avoid overlap) and mean log titers. While there is a good deal of variation in individual titers, as expected, there are trends in the mean log titers that we capture with the APC models. Correlations between the log titers are given in Figure D. Titers to viruses within the same antigenic cluster are highly correlated (above 0.9 for PE09 cluster and above 0.8 for C09 cluster).

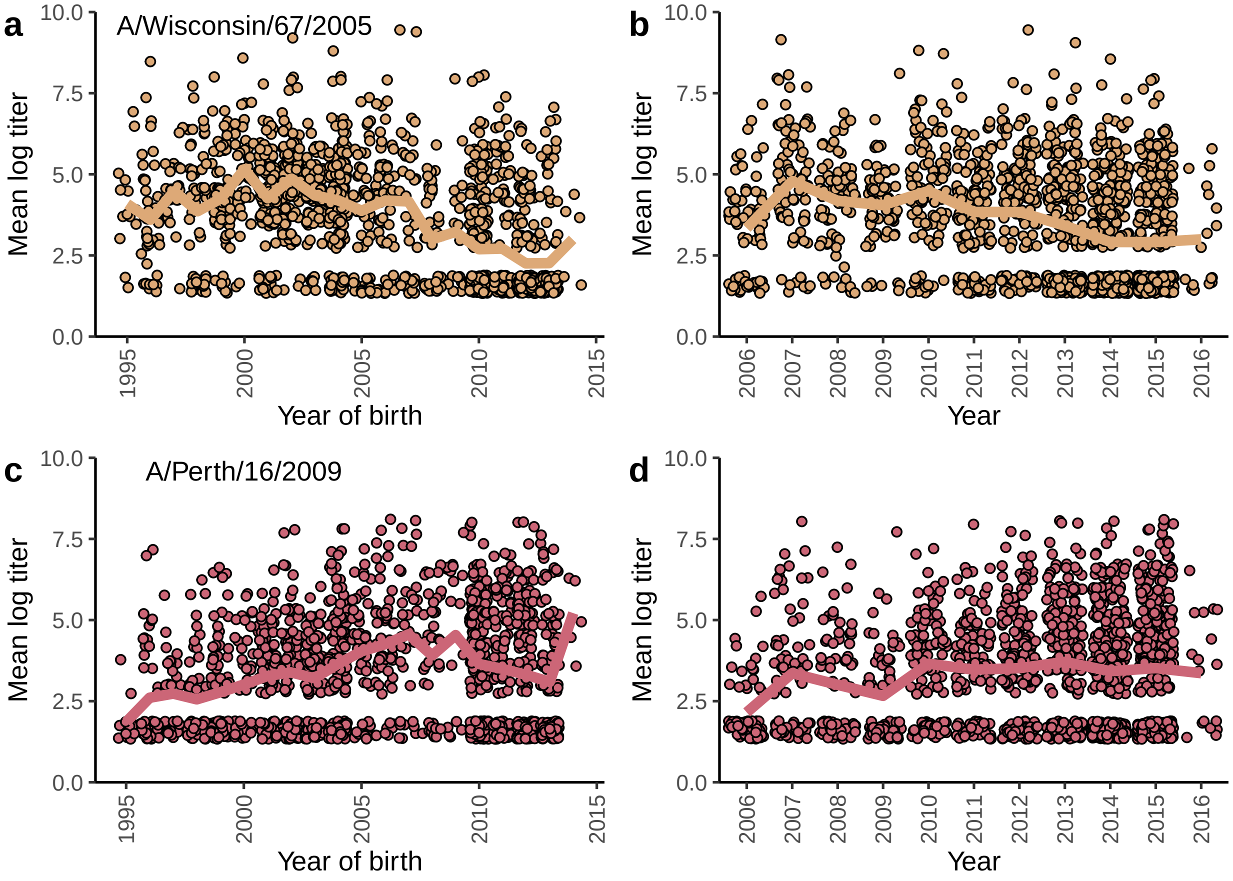

Figure B: **H3N2 log titers and mean log titers by birth cohort and calendar year.** Individual log titers are jittered to avoid overlaps. a, b) A/Wisconsin/67/2005; c, d) A/Perth/16/2009; e, f) A/Victoria/361/2011; g, h) A/Texas/50/2012.

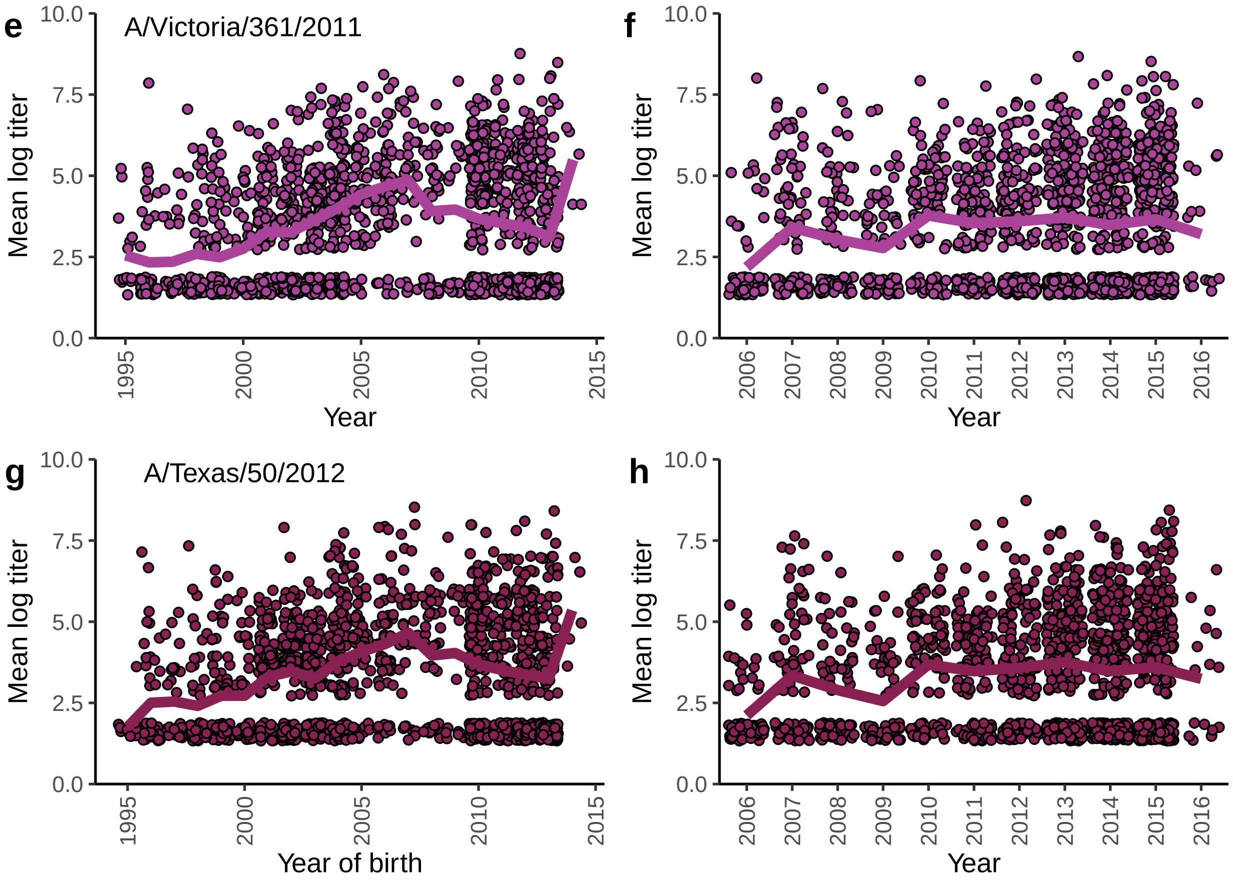

Figure B: cont.

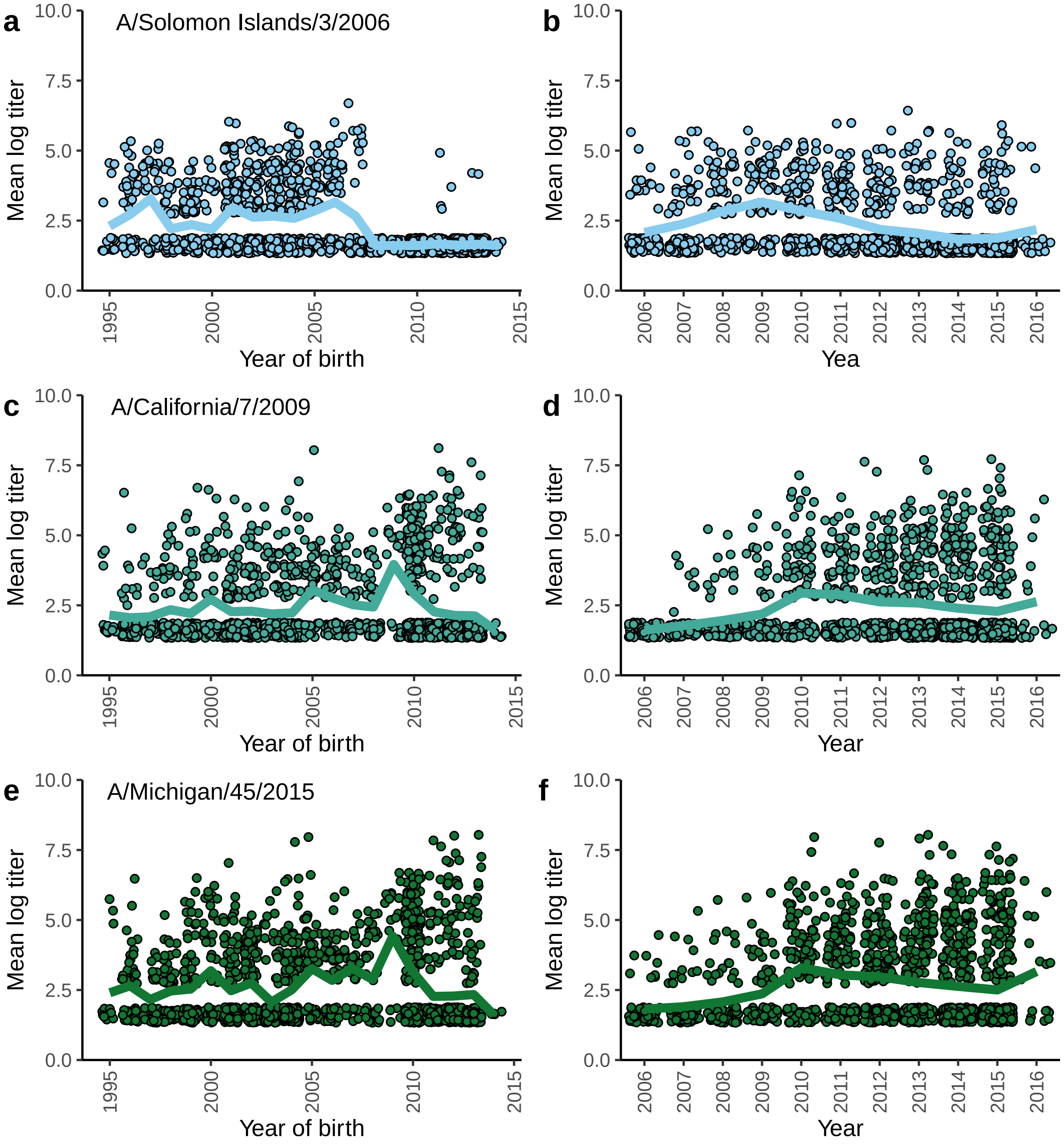

Figure C: **H1N1 log titers and mean log titers.** Individual log titers are jittered to avoid overlaps. a, b) A/Solomon Islands/3/2006; c, d) A/California/7/2009; e, f) A/Michigan/45/2015.

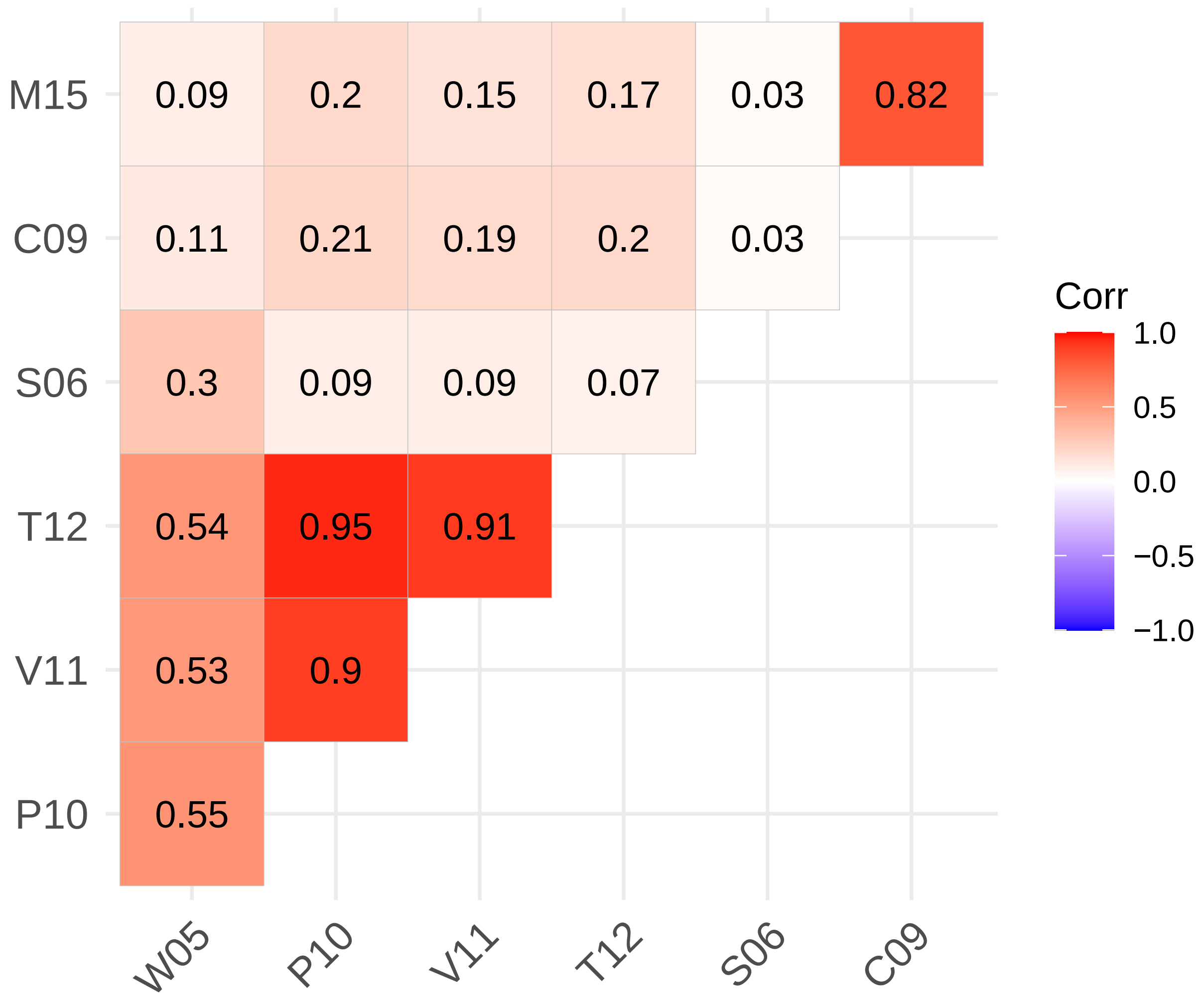

Figure D: **Correlation of influenza A titers by virus within individuals.**

Comparison of APC models

In Tables B and C, we present a variety of model fit metrics for each of the considered APC models. These metrics validate the choice of modeling period and cohort as the drivers of population-level antibody titers. Although the strain-specific models can achieve a better fit to the data (as described by higher R^2, lower root mean squared error ($\sqrt{\text{MSE}}$), and lower negative log-likelihood (NLL)), the subtype-specific models achieve nearly the same fits more parsimoniously (as evidenced by a lower Schwarz Information Criterion (SIC)). Among the subtype-specific models, defining cohort as age at cluster introduction achieves a better fit than either age at strain introduction or year of birth.

Table B: **Comparison of APC models for H3N2 log-titers**. Models are compared by degree of freedom (df), R^2, root mean squared error ($\sqrt{\text{MSE}}$), negative log-likelihood (NLL), number of parameters (N_{par}), and Schwarz Information Criterion (SIC). In the strain specific models, all specifications of cohort are equivalent.

Model	df	R^2	$\sqrt{\text{MSE}}$	NLL	N_{par}	SIC
**Subtype-specific models**						
Age	6348	0.01	1.75	12582.2	7	25225.7
Period	6341	0.03	1.73	12515.5	14	25153.6
Cohort: birth year	6347	0.05	1.71	12436.5	8	24943.1
Cohort: strain introduction	6347	0.11	1.66	12251.1	8	24572.2
Cohort: cluster introduction	6347	0.12	1.65	12208.2	8	24486.5
Age + Period	6338	0.04	1.72	12480.0	17	25108.9
Age + Cohort: birth year	6344	0.06	1.71	12416.3	11	24929.0
Age + Cohort: strain introduction	6344	0.11	1.66	12237.1	11	24570.6
Age + Cohort: cluster introduction	6344	0.12	1.65	12184.1	11	24464.4
Period + Cohort: birth year	6337	0.07	1.69	12365.0	18	24887.7
Period + Cohort: strain introduction	6337	0.13	1.64	12174.3	18	24506.2
Period + Cohort: cluster introduction	6337	0.14	1.63	12134.3	18	24426.2
**Strain-specific models**						
Age	6339	0.06	1.71	12424.6	16	24989.3
Period	6311	0.06	1.70	12405.8	44	25196.9
Cohort	6335	0.12	1.65	12202.2	20	24579.6
Age + Period	6299	0.10	1.66	12254.6	56	24999.5
Age + Cohort	6323	0.13	1.64	12180.6	32	24641.5
Period + Cohort	6295	0.14	1.63	12123.4	60	24772.2

Table C: **Comparison of APC models for H1N1 log-titers**. Models are compared by degree of freedom (df), R^2, root mean squared error ($\sqrt{\text{MSE}}$), negative log-likelihood (NLL), number of parameters (N_{par}), and Schwarz Information Criterion (SIC). In the strain specific models, all specifications of cohort are equivalent.

Model	df	R^2	$\sqrt{\text{MSE}}$	NLL	N_{par}	SIC
**Subtype-specific models**						
Age	4757	0.04	1.30	7999.2	6	16049.2
Period	4750	0.06	1.28	7928.7	13	15967.4
Cohort: birth year	4756	0.05	1.29	7972.7	7	16004.8
Cohort: strain introduction	4756	0.06	1.28	7939.7	7	15938.7
Cohort: cluster introduction	4756	0.08	1.27	7878.3	7	15815.8
Age + Period	4747	0.08	1.27	7888.0	16	15911.4
Age + Cohort: birth year	4753	0.05	1.29	7958.9	10	16002.4
Age + Cohort: strain introduction	4753	0.07	1.28	7925,7	10	15936.0
Age + Cohort: cluster introduction	4753	0.09	1,26	7863.1	10	15811.0
Period + Cohort: birth year	4746	0.08	1.27	7882.0	17	15908.0
Period + Cohort: strain introduction	4746	0.10	1.26	7842.0	17	15827.9
Period + Cohort: cluster introduction	4746	0.12	1.24	7784.5	17	15713.0
**Strain-specific models**						
Age	4751	0.06	1.28	7951.5	12	16004.7
Period	4730	0.10	1.26	7847.9	33	15975.2
Cohort: birth year	4748	0.09	1.26	7860.3	15	15847.7
Cohort: strain introduction	4748	0.09	1.26	7866.4	15	15859.8
Cohort	4748	0.09	1.26	7868.6	15	15864.1
Age + Period	4721	0.12	1.23	7770.8	42	15897.4
Age + Cohort	4739	0.10	1.25	7839.7	24	15882.7
Period + Cohort	4718	0.14	1.23	7734.1	45	15849.3

Validating the estimated cohort effects

In order to demonstrate that the cohort effects estimated in the main text are reasonable representations of the data, we first provide the raw mean log titers for each strain by age at the year of cluster introduction identified in the main text (Figure E). These data do not adjust for calendar year. The models in the main text do capture the shape of the effects seen in the data. Note that the spike in mean log titer for the three viruses in the PE09 cluster is driven by the small number of individuals sampled in 2016, as seen in Figure B.

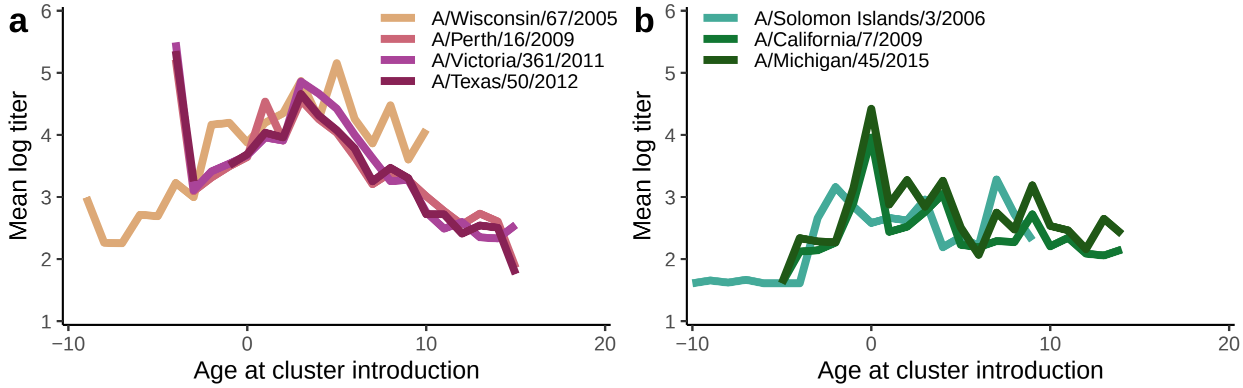

Figure E: **Cohort effects in the raw data.** a) Mean log titer of the H3N2 strains by age at cluster introduction. b) Mean log titer of the H1N1 strains by age at cluster introduction.

Second, we provide bootstrap sensitivity analysis of the H3N2 and H1N1 subtype cohort effects. We sample each data point with replacement to generate a bootstrap data set on which we estimate the cohort effects. The results for 100 bootstrap samples are given below in Figure F. These plots demonstrate that our results are not driven by outliers in the data.

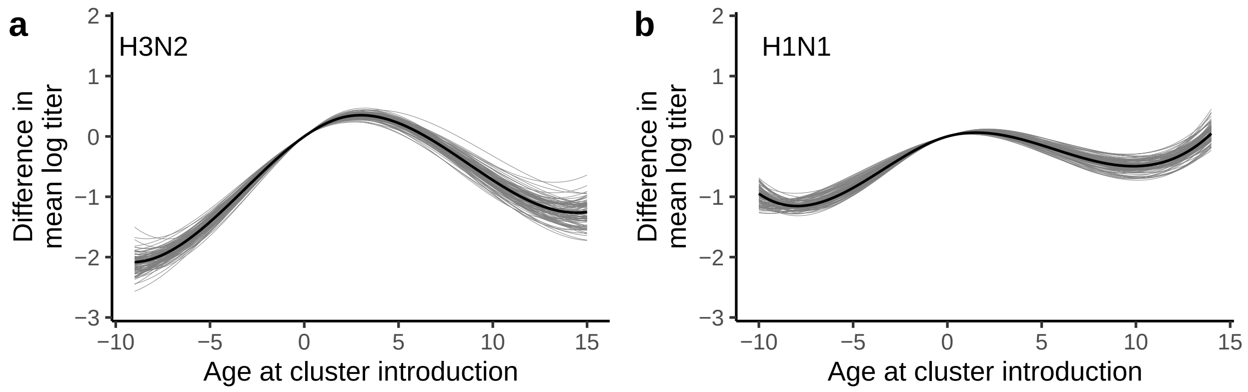

Figure F: **Bootstrap cohort effects for a) H3N2 and b) H1N1.** Individual bootstrap estimates are in grey, and the estimate for the original data set is in black.

Interpreting population-level cohort effects

In order to interpret the population-level cohort effects, we plotted the fraction of the children enrolled prior to age 1 that had antibodies to each strain (i.e., titers greater than 1:5) as function of time since cluster introduction. We see that this fraction decays over time (Figure G), indicating that the time to first infection increases with time since cluster introduction. A larger fraction of children has antibodies to H3N2 than H1N1. However, if children do not have antibodies prior to age 1, they eventually catch up, presumably once they have an infection by a strain in the cluster. At the population-level, we can distinguish between the trajectories of children with and without early-life antibodies (Figure H). Although these plots show titers slowly increasing for the children without initial titers, this pattern is driven by a changing fraction of children with and without titers (as individuals are infected) rather than a population-level rise. In these data, this catch-up takes 2–4 years for H3N2 and 4–5 for H1N1, consistent with the less frequent circulation of H1N1 compared to H3N2. We do not include A/Solomon Islands/3/2006 in this analysis because there were few children in this subset (all born 2011 or after) had any titers to this strain.

Looking at the larger sample of participants, we plot the mean log-titer for each strain, grouped by which antigenic cluster each individual was born into (Figure J). Children in the birth cohorts shortly preceding the antigenic cluster introduction (those with the highest titers overall) had cross-reactive antibodies prior to circulation of the first virus in the new antigenic cluster. Children in earlier birth cohorts have lower titers overall because they initially begin with lower titers but then have titers boosted around the time of circulation. Children born while strains of in the same antigenic cluster as a given strain are circulating appear to maintain relatively high levels of HAI antibodies cross-reactive to the given strain, at least as long as strains in that cluster are circulating. Children born after strains in a given antigenic cluster have stopped circulating appear had few cross-reactive HAI antibodies to strains in that cluster.

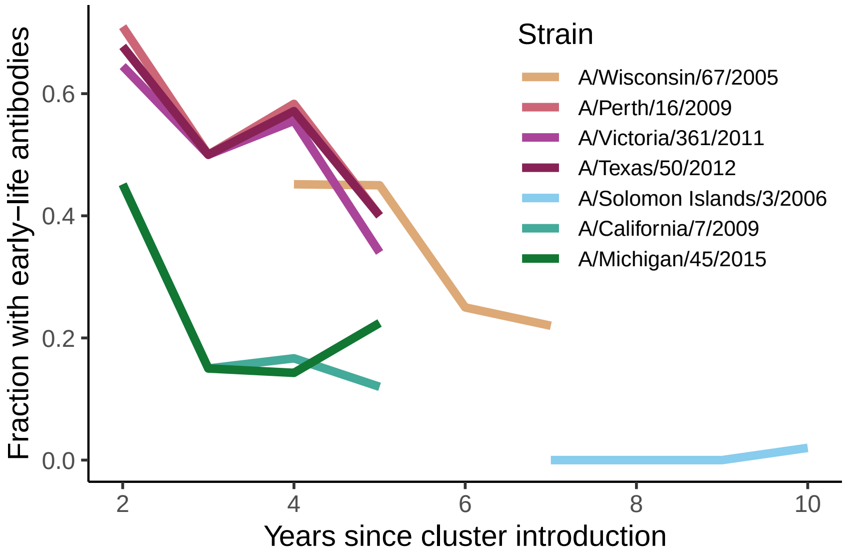

Figure G: **Fraction of children enrolled prior to age 1 who had antibody titers to the given strain as a function of the time since cluster introduction.**

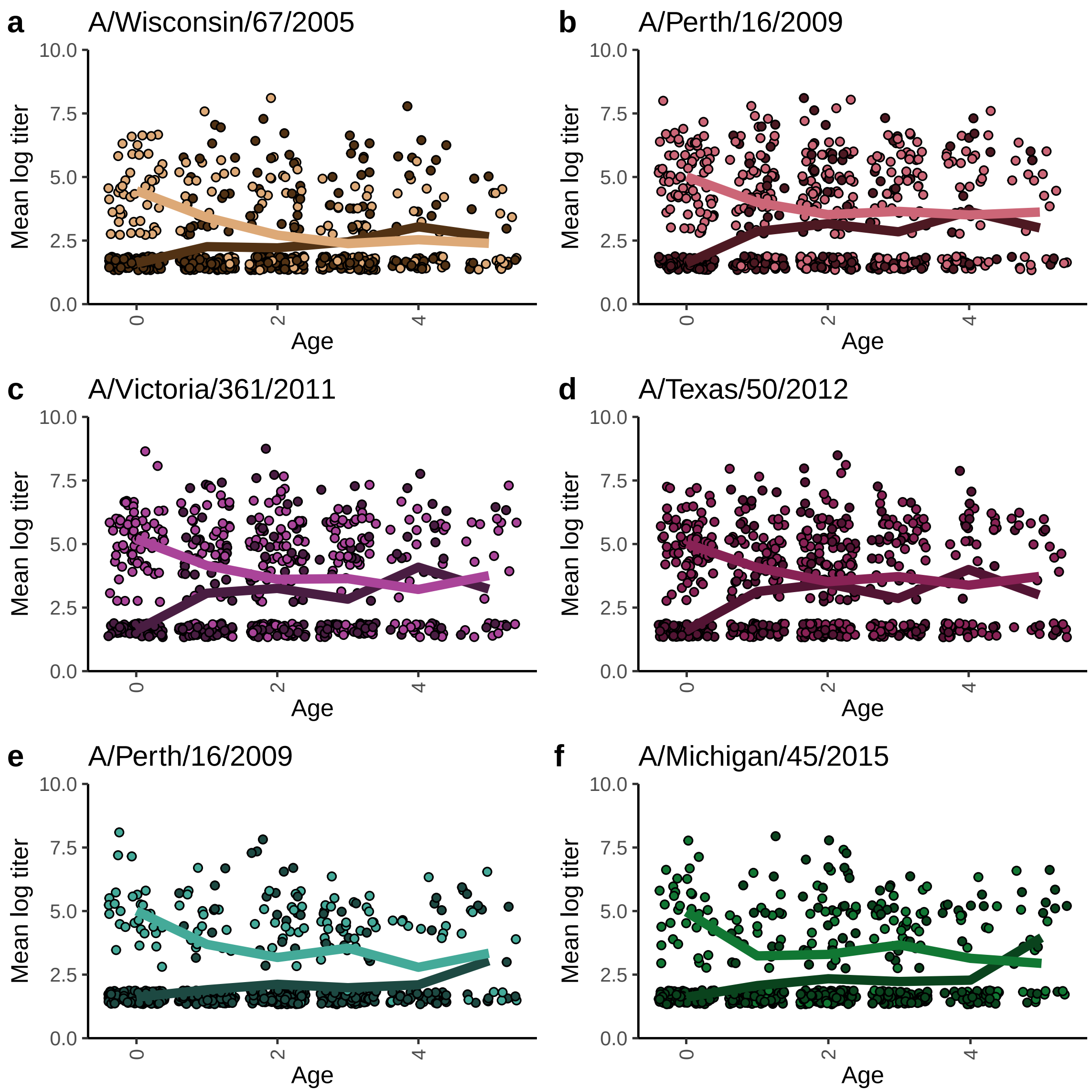

Figure H: **Population-level average mean log antibody titer trajectories.** Trajectories for children enrolled prior to age 1, distinguishing between those who had antibodies to the given strain prior to age 1 and those that did not.

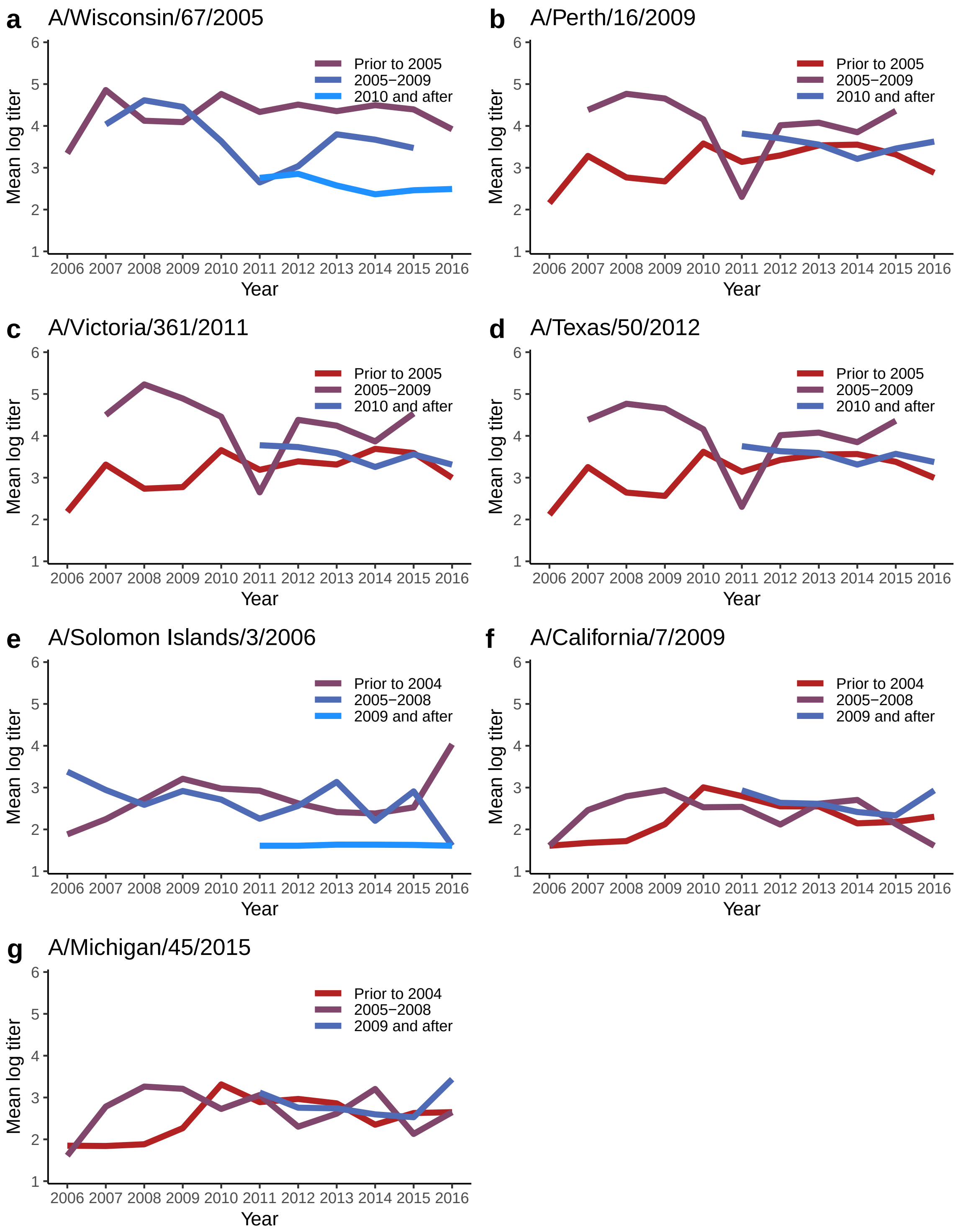

Figure I: **Mean log titer in each year for each strain, stratifying the population by birth cohort relative to the change in antigenic cluster of the circulating virus.** Red indicates those born more than one antigenic cluster before the given strain's cluster, purple indicates those born in the antigenic cluster just prior to the given strain's cluster, dark blue indicates those born in years the given strain's cluster was circulating, and light blue indicates those born in years after the given's strains cluster was no longer circulating.

Phylogentic analysis

To confirm the likely antigenic cluster of the H3N2 viruses circulating in Nicaragua in 2007 and 2010, we implemented a maximum-likelihood phylogentic tree of the H3 segment of viruses isolated 2005–10 in Nicaragua and the US, as well as vaccine strains. The Nicaragua strains from 2007 are BR07-like and those from 2010 are PE09-like, which substantiates our hypothesis that the strains from those years were from distinct antigenic clusters.

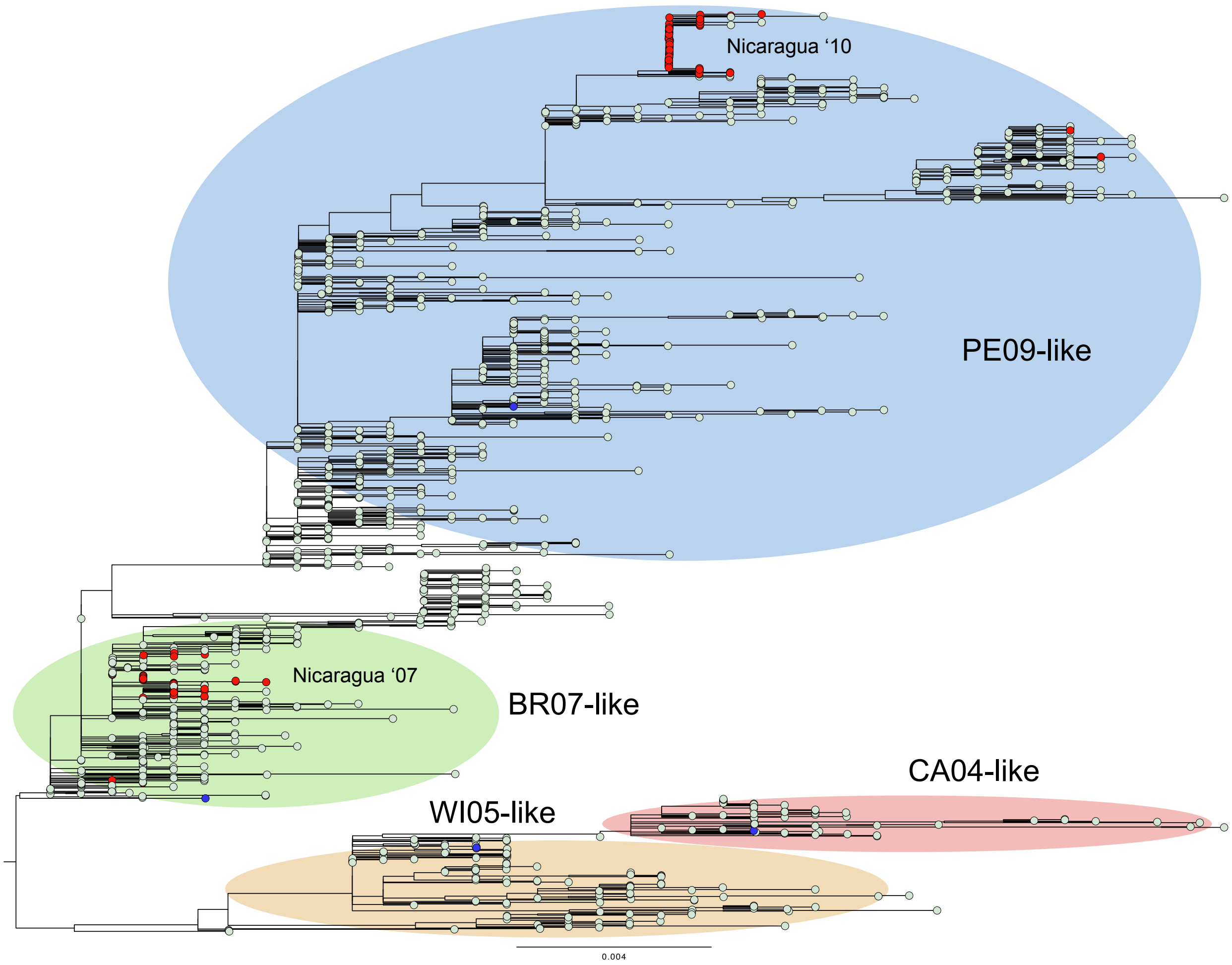

Figure J: **Maximum likelihood tree of H3 proteins, 2005–10.** Nicaraguan viruses are in red, US viruses are in light green, and vaccine viruses are in blue. The Nicaragua strains from 2007 are BR07-like, and those from 2010 are PE09-like.
